# Precision medicine applied to metastatic colorectal cancer using tumor-derived organoids and in-vitro sensitivity testing: a phase 2, single-center, open-label, and non-comparative study

**DOI:** 10.1186/s13046-023-02683-4

**Published:** 2023-05-05

**Authors:** Lars Henrik Jensen, Silvia Regina Rogatto, Jan Lindebjerg, Birgitte Havelund, Cecilie Abildgaard, Luisa Matos do Canto, Chris Vagn-Hansen, Claus Dam, Søren Rafaelsen, Torben Frøstrup Hansen

**Affiliations:** 1 Department of Oncology, University Hospital of Southern Denmark, Lillebaelt Hospital, Beriderbakken 4, Vejle, 7100 Denmark; 2 Danish Colorectal Cancer Center South, University Hospital of Southern Denmark, Vejle, Denmark; 3 Institute of Regional Health Research, University of Southern Denmark, Odense, Denmark; 4 Clinical Genetics Department, University Hospital of Southern Denmark, Lillebaelt Hospital, Vejle, Denmark; 5 Department of Pathology, University Hospital of Southern Denmark, Lillebalt Hospital, Vejle, Denmark; 6 Department of Radiology, University Hospital of Southern Denmark, Lillebalt Hospital, Vejle, Denmark

**Keywords:** Metastatic colorectal cancer, Drug screening, Tumor-derived organoids, Historical controlled phase II trial

## Abstract

**Background:**

Patients with colorectal metastatic disease have a poor prognosis, limited therapeutic options, and frequent development of resistance. Strategies based on tumor-derived organoids are a powerful tool to assess drug sensitivity at an individual level and to suggest new treatment options or re-challenge. Here, we evaluated the method’s feasibility and clinical outcome as applied to patients with no satisfactory treatment options.

**Methods:**

In this phase 2, single-center, open-label, non-comparative study (ClinicalTrials.gov, register NCT03251612), we enrolled 90 patients with metastatic colorectal cancer following progression on or after standard therapy. Participants were 18 years or older with an Eastern Cooperative Oncology Group performance status of 0–2, adequate organ function, and metastasis available for biopsy. Biopsies from the metastatic site were cultured using organoids model. Sensitivity testing was performed with a panel of drugs with proven activity in phase II or III trials. At the discretion of the investigator considering toxicity, the drug with the highest relative activity was offered. The primary endpoint was the proportion of patients alive without disease progression at two months per local assessment.

**Results:**

Biopsies available from 82 to 90 patients were processed for cell culture, of which 44 successfully generated organoids with at least one treatment suggested. The precision cohort of 34 patients started treatment and the primary endpoint, progression-free survival (PFS) at two months was met in 17 patients (50%, 95% CI 32–68), exceeding the pre-defined level (14 of 45; 31%). The median PFS was 67 days (95% CI 51–108), and the median overall survival was 189 days (95% CI 103–277).

**Conclusions:**

Patient-derived organoids and in-vitro sensitivity testing were feasible in a cohort of metastatic colorectal cancer. The primary endpoint was met, as half of the patients were without progression at two months. Cancer patients may benefit from functional testing using tumor-derived organoids.

**Trial registration:**

ClinicalTrials.gov, register NCT03251612.

**Supplementary Information:**

The online version contains supplementary material available at 10.1186/s13046-023-02683-4.

## Highlights

Metastatic colorectal cancer patients (*N* = 90) were enrolled in a phase 2 study.

Drug assays were successfully tested in tumor derived organoids of metastatic colorectal cancer patients.

Sensitivity drug tests performed in tumor derived organoids pointed out specific treatment for individual patients.

A total of 34 patients initiated the treatment according to the drug assay results.

Progression-free survival at two months was met in 17 of 34 patients exceeding the pre-defined minimal relevant difference to historical controls from randomized trials.

**Supplementary Information:**

The online version contains supplementary material available at 10.1186/s13046-023-02683-4.

## Introduction

Patients with metastatic or unresectable colorectal cancer have limited options for cancer directed treatment [1]. According to SEER Cancer Statistics Review 1975–2015, 20–22% of colorectal cancer patients have metastatic disease at diagnosis, and 50–60% develop metastasis during the disease course [2]. Re-challenge with a previous line of well tolerated chemotherapy may be an alternative [3, 4], or new drugs or combination therapy can be considered. However, according to the ESMO Magnitude of Clinical Benefit Scale, third-line drugs such as regorafenib and trifluridine tipiracil (TAS-102) have no substantial clinical benefit [5]. The median survival gained is only one or two months, and considering the side effects, palliative care would be the best option for most of these patients. We hypothesize that a low average benefit from an available drug may become substantial if patients are tested and proven resistant to the drug and, therefore, excluded from the treatment. A clinically applicable method is needed to allocate the individual patient to the available treatment with the highest chance of effect.

One approach to the individual selection of drugs is in vitro sensitivity testing of patient-derived tumor organoids (TDOs). Colorectal cancer cells expanded in three-dimensional (3D) cultures retain their molecular subtype and are amenable to high-throughput drug screens [6]. The small amount of tissue obtained from biopsies of metastatic colorectal cancer patients is suitable for generating TDOs [7]. Short-term 3D cell cultures have proven feasible with a similar genomic profile of their derived tumor [8] which allows clinical application in cancer patients with a progressive disease and limited time from biopsy to the start of treatment.

In the present trial, we assessed metastatic colorectal cancer patients’ feasibility and clinical outcome using individualized in vitro tumor response tests. The patients with no satisfactory treatment options underwent biopsies of the metastatic site to generate TDOs. Next, sensitivity testing assays were performed in those TDOs using selected drug panels. Based on these results, an individualized anti-cancer treatment was conducted. Herein, we report the clinical results of patients allocated to precision treatment, the precision cohort.

## Patients and methods

### Study design and participants

This phase 2, single-center, open-label, historically controlled trial enrolled patients with non-resectable metastases from colorectal cancer. The selection criteria included patients at age ≥ 18 years, Eastern Cooperative Oncology Group performance status (PS) = 0–2, adequate organ function with absolute neutrophil count ≥ 1.5 × 10^9^/L, thrombocytes ≥ 100 × 10^9^/L, bilirubin ≤ 1.5 x upper normal value, alanine aminotransferase ≤ 3 x upper normal value, and calculated or measured renal glomerular filtration rate at least 30 mL/min. Other inclusion criteria were previous exposure, intolerance or contraindications to standard systemic therapy (oxaliplatin, irinotecan, 5-fluorouracil or derivatives (5FU), bevacizumab, and if indicated, panitumumab or cetuximab) and documented evidence of progression according to the Response Evaluation Criteria in Solid Tumors (RECIST) version 1.1. Pregnant or breastfeeding women were excluded, as were patients with incapacity, frailty, disability, substantial comorbidity, other active malignancy, or ongoing systemic anti-cancer therapy.

All patients provided written informed consent, and the trial was conducted following the guidelines for Good Clinical Practice and the Declaration of Helsinki. The Danish Medicines Agency (EudraCT no 2017-000456-26) and The Regional Committee on Health Research Ethics for Southern Denmark (S-2017-0028) approved the protocol. In February and September 2019, amendments adding vemurafenib, temozolomide, binimetinib, and encorafenib were approved. The trial was prospectively registered with ClinicalTrials.gov (NCT03251612) on August 16, 2017, before the inclusion of the first patient. The protocol sponsor reviewed the study feasibility, biopsy, logistics, and in vitro testing after enrolling ten patients to recommend continued recruitment, protocol amendment, or retraction of the study.

### Sample collection

The enrolled patients underwent a biopsy of the metastasis (liver in > 90% of cases) based on the safety of the procedure and the accessibility of the lesion. All available imaging was evaluated to select active metabolic metastases with size progression, activity on fluorodeoxyglucose positron emission tomography, or diffusion restriction on magnetic resonance imaging, but only a computerized tomography scan (CT) was mandatory. Prior to the biopsies, all patients had a blood sample drawn to evaluate hemoglobin (Hgb) and coagulation parameters. The biopsy levels were set at Hgb concentration > 5 mmol/L, coagulation factor normal test (coagulation factor II, VII, X) > 0.4, activated partial thromboplastin time < 40 s. and thrombocytes > 40 × 10^9^/L.

Image-guided biopsies were performed by an experienced radiologist using a SuperCore™ Semi-Automatic Biopsy Instrument (Argon Medical Devices, Inc.,1445 Flat Creek Road Athens, Texas 75,751 USA). An Aplio i800 ultrasound unit (Canon Medical Systems Corporation, 1385 Shimoishigami, Otawara-shi, Tochigi 324–8550, Japan) with a needle guide system (Verza™ Guidance System, CIVCO Medical Instruments Co., Inc. 102 First Street South, Kalona, IA, USA) was used for liver biopsy. CT-guided biopsies were performed with a CT-guided “beam-through” technique using a 64-slice CT scanner (Phillips Brilliance 64, Eindhoven, Netherlands). Re-biopsy was allowed if the patient was in good clinical performance. One to three (preferably) 18G to 16G (preferably) biopsies were sampled in a collection tube containing sterile phosphate buffered saline, 500 U/mL penicillin, 500 µg/mL streptomycin, and 5 mg/mL amphotericin. In parallel, a biopsy was formalin-fixed and paraffin-embedded, and a 4-micron section stained with Hematoxylin-Eosin was reviewed to confirm the presence of viable tumor cells.

### Patient-derived tumor organoids (TDOs)

The biopsies collected in buffered saline were processed within three hours. The tissue was dissociated mechanically with needles into small fragments (1 mm^3^), followed by enzymatic digestion with collagenase II (Gibco, Thermo Fisher Scientific, Waltham, USA). The released and washed cells were plated in BD Matrigel ™Basement Membrane Matrix (Corning, New York, USA) and cell StemPro™ growth media (Gibco, Thermo Fisher Scientific, Waltham, USA) (1:1) and incubated at 37^o^C and 5% CO_2_. The cell growth medium is composed of AdvDMEM/F-12, GlutaMAX™ medium, bovine serum albumin 25%, StemPro®hESC Supplement (Thermo Fisher Scientific, Waltham, USA), added with FGF (10 µg/mL) (Thermo Fisher Scientific, Waltham, USA), 2-Mercaptoethanol (Thermo Fisher Scientific, Waltham, USA), Penicillin/Streptomycin (10,000U/ 10 mg/mL), Gentamycin, and Amphotericin (2.5 µg/mL each) (Sigma-Aldrich, St. Louis, MO, USA). To increase the chance of TDO formation, we added 10µM ROCK Inhibitor (Y-27,632) (Sigma Aldrich, St. Louis, Missouri, USA) for the first two passages. The medium was changed every 2–3 days, and the PDOs were used for drug sensitivity testing (passage four or lower). Cultures were checked for mycoplasma contamination as a routine protocol (MycoAlert Mycoplasma Detection Kit, Lonza).

### Histological characterization

The second half of the biopsies of mCRC uncultured and TDOs (10 to 18 days) were fixed and histologically evaluated using Tissue-Teck VIP 6AI Tissue Processor (Sakura Finetek, Japan). Both samples were stained with hematoxylin-eosin to confirm the presence of tumor cells. The CDX2 (clone AMT 28, NovoCastra; 1:50) expression was evaluated using the Benchmark Ultra automated instrument (Ventana Medical Systems, Roche, Tucson, USA).

### Drug sensitivity testing in patient-derived organoids

Drug sensitivity testing was performed in TDOs generated from metastatic colorectal biopsies using the IndiTreat® platform from 2cureX laboratories (https://www.2curex.com/). IndiTreat® is a family of CE-IVD tests developed to predict the response to different drug regimens. Briefly, the IndiTreat® drug sensitivity test exposes the TDOs to the drugs for seven days, and cell growth is compared to untreated TDOs (negative controls) from the same patient. The drug panels were established based on the literature showing effect in phase II or III trials added with capabilities of the IndiTreat® drug sensitivity test (2cureX, Copenhagen, Denmark) (Supplementary Table S1). The following drugs were included in the panels as monotherapy: 5FU, regorafenib, TAS-102, sorafenib, olaparib, and epirubicin. Other drugs were included or combined, as described below: 5FU, oxaliplatin, irinotecan, cetuximab, vinorelbine, gemcitabine, vemurafenib, temozolomide, encorafenib, and binimetinib. The number of drugs tested depended on the number of TDOs available in each patient. Supplementary Table 1 details the drug panels tested in TDOs.

Cell growth was measured by taking brightfield images on day zero and visualizing living cells on day seven using a fluorescent stain (CyQUANT Cell Proliferation Assay, ThermoFisher Scientific Inc.). Images from days zero and seven were analyzed using the artificial intelligence image analysis algorithm IndiNet™. IndiNet™ quantifies the area of TDOs in both brightfield (day zero) and fluorescence (day seven) images and calculates the relative drug induced growth inhibition by comparing treated with untreated TDOs from the same patient (Supplementary Figure S1A). The relative growth inhibition for each treatment was compared to similar results from a panel of reference patients with metastatic colorectal cancer at the same disease stage. The relative growth inhibition of 100 reference patients followed a normal distribution. A minimum of six replicates was used in the drug testing with at least four TDOs. The relative treatment sensitivities were classified as sensitive, very sensitive, low, and very low (Supplementary Figure S1B). In cases with a limited number of TDOs, the drugs tested were prioritized by the treating physician based on the clinical evaluation, such as previous resistance or toxicity profile.

### Patients’ treatment and evaluations

The investigator reviewed the sensitivity test report performed in 3D cells, considered specific contraindications and adverse events relevant to the individual patient, and suggested the drug or combination of drugs with the highest relative activity. Patients were only treated with the IndiTreat-guided drug(s) if the test for the specific treatment showed sensitivity in one of the two most sensitive groups. Patient preference, e.g., oral treatment or convenience, was met in the case of clinical equipoise. Standard regimens were administered according to institutional practice for irinotecan and 5FU (FOLFIRI), oxaliplatin and 5FU (FOLFOX), irinotecan, oxaliplatin and 5FU (FOLFOXIRI), and combinations with panitumumab or bevacizumab. For non-standard regimens, the treatment followed the summary of product characteristics. Drugs not explicitly approved for colorectal cancer were administered to the patients using the dosage recommended to other cancer patients.

The combination of vinorelbine and capecitabine was given in 3-weekly cycles as oral vinorelbine 80 mg/m^2^ (after a first cycle at 60 mg/m^2^) on day 1 and day 8 together with capecitabine 1000 mg/m^2^ (750 if age ≥ 65 years) twice daily on days 1–14 [9] in 3-weekly cycles. Gemcitabine 1000 mg/m^2^ i.v. was given in 2-weekly cycles on day 1 together with capecitabine 1000 mg/m^2^ twice daily on days 1–7 [10]. Vemurafenib 960 mg twice daily was given with irinotecan 180 mg/m^2^ i.v. and cetuximab 500 mg/m^2^ i.v. every two weeks. Temozolomide 150 mg/m^2^ on days 1–5 was given with irinotecan 100 mg/m^2^ on days 1 and 15 every 28 days [11]. Encorafenib 300 mg orally once daily was combined with standard cetuximab (institutional practice 500 mg i.v. bi-weekly). Binimetinib 45 mg twice daily orally was added only if sensitivity was proved [12].

Tumor response was assessed with CT according to RECIST 1.1. If at the time of the first treatment dose, the baseline scan was older than four weeks, a new CT scan was performed and then repeated every 8 weeks until disease progression. Adverse events were reviewed according to the Common Terminology Criteria for Adverse Events, version 4, before and during treatment.

### Outcomes

Treatment response and cancer progression were assessed using RECIST 1.1. The primary endpoint was defined as the proportion of patients alive without disease progression at two months. Secondary endpoints were progression-free survival (PFS) (calculated from the start of treatment start to the date of first documented progression or the death of any cause) and overall survival (OS) (recorded from the first day of treatment until the death of any cause) using the Kaplan-Meier method. The treatment response of each patient was assigned to the following categories: complete, partial, stable disease, and progressive disease.

### Statistical analysis

The sample size was based on Simon’s optimal two-stage design and admissible design from Jung et al. for Phase II single arm clinical trials [13, 14]. In previous last-line randomized trials with a placebo comparator, 20% of the patients in placebo groups were alive and progression-free at the first evaluation [15, 16]. It was deemed of clinical interest if the rate of PFS at the first evaluation was improved to 0.40. If the one-sided type I error rate is 0.05, power 0.9, and a minimax design is chosen, up to 45 patients should be enrolled. In stage 1, 24 patients were accrued, and the trial would be stopped if less than five were progression-free at the first evaluation. Enrollment would continue during the evaluation. If the stopping criterion was not met, another 21 patients would be enrolled to a total of 45. The study would be positive with at least 14 patients with PFS at the first evaluation. It was estimated that half of the patients would have a successful sensitivity test resulting in 90 patients. Statistical analyses were performed using STATA software version 16 (StataCorp, College Station, Texas 77,845 USA).

## Results

A planned feasibility assessment was done after the enrollment of 10 patients. Re-biopsies were necessary in several cases, and 19 biopsy sessions were performed using ultrasound (n = 14), CT (n = 3), and sigmoidoscopy (n = 2). The biopsy, TDOs generation, and sensitivity testing were successful in seven patients, with a median time from biopsy collection to drug testing results of 34 days (range 19–50) [17]. Since, according to the protocol, at least half of the 10 patients were to be offered treatment, enrollment continued.

**Fig. 1:**
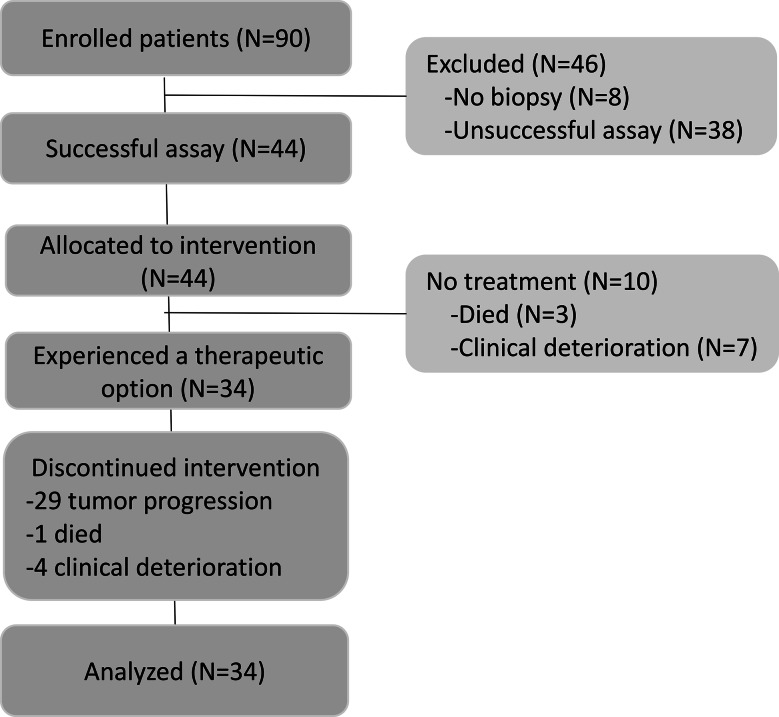
Study design showing the precision cohort as the group of patients who received treatment for metastatic colorectal cancer based on successful biopsy and drug screening performed in patient-derived tumor organoids

The planned number of 90 patients was enrolled for three years in one center (September 25, 2017 to September 14, 2020). The mCRC biopsies and their corresponding derived tumor organoids were evaluated by hematoxylin-eosin staining to confirm the presence of tumor cells (Supplementary Figure S2). The patient flow is shown in Fig. 1. Among the 34 patients that received the treatment, 31 biopsies were collected from the liver and three in other sites (1 lung, 1 peritoneum, and 1 in adrenal gland). Moreover, nine patients had one metastatic site, 12 presented two metastatic sites, 10 patients had three metastatic sites, and three had four metastatic sites. Re-biopsies of the same metastatic site were collected if the first (n = 9) or second (n = 2) biopsy failed. The biopsy was successful collected in 82 patients, of which 44 had TDOs generated for sensitivity testing.

All 44 patients had a significant number of TDOs, and the sensitivity test was successful with at least one treatment suggested. Before the results were obtained, three patients died and seven had clinically deteriorated, leaving 34 patients who initiated treatment within a median of 51 days from enrollment (interquartile range, IQR = 39–63). This cohort was defined as the precision cohort, and subsequent results are detailed for these patients. Patient characteristics at baseline are shown in Table 1. The patients were followed until death, and five were alive at the analysis time. Median follow-up was 4.5 months (IQR = 3.2–9.5 months).

**Table 1:** Clinical features of 34 patients enrolled in the study

Characteristics	Number (%)
Age in years (Median - IQR)	61 (54–71)
Sex	
Female	20 (59)
Male	14 (41)
Race	
White	34 (100)
Body surface area, m^2^ (Median -IQR)	1.9 (1.8–2.1)
Performance status	
0	25 (74)
1	9 (26)
2	0 (0)
Primary tumor	
Right colon	7 (21)
Left colon	18 (53)
Rectum	9 (26)
*KRAS* (*N* = 34), *NRAS* (*N* = 27) or *BRAF* (*N* = 31)^a^	
All wild-type	8 (24)
Mutation	26 (76)
Mismatch repair	
Proficient	33 (97)
Deficient	0 (0)
Unknown	1 (3)
Primary tumor removed	
Yes	25 (74)
No	9 (26)
Number of previous regimens for metastatic disease, including rechallenge (Median - IQR)	3 (2.25-3)
Current extent of disease, metastatic sites^b^	
Liver	32 (49)
Lung	22 (65)
Lymph nodes	8 (24)
Other	13 (38)

^a^n indicates the number of patients who had the analysis performed. ^b^ Patients presented more than one metastatic site. IQR: interquartile range

Based on the drug testing results on TDOs, nine different regimens were administered for 34 metastatic colorectal cancer patients. Supplementary Table S2 details the drug panels tested, and the sensitivity response of each drug observed in the TDOs from 34 patients. The most frequently suggested treatment was gemcitabine-capecitabine followed by vinorelbine-capecitabine and TAS-102. Less commonly given drugs were temozolamide-irinotecan, FOLFIRI, regorafenib, sorafenib, epirubicin, and olaparib (Table 2). The median time between the first and last dose of chemotherapy was 54 days (IQR = 36–106). No patient in this cohort received oxaliplatin, cetuximab, vemurafenib, encorafenib, or binimetinib treatment.

**Table 2:** Based on patient-derived tumor organoids formation and sensitivity test, 34 patients initiated the treatment. Treatment regimens and number of patients alive and without progression (PFS) at 2 months

Treatment	Number of patients treated (*N* = 34)	Number of patients with PFS at 2 months (*N* = 17)
Gemcitabine-capecitabine	12	6
Vinorelbine-capecitabine	6	3
TAS-102	5	1
Temozolamide-irinotecan	3	2
FOLFIRI*	2	2
Regorafenib	2	0
Sorafenib	2	2
Epirubicin	1	1
Olaparib	1	0

* Bevacizumab was added in one case

**Fig. 2:**
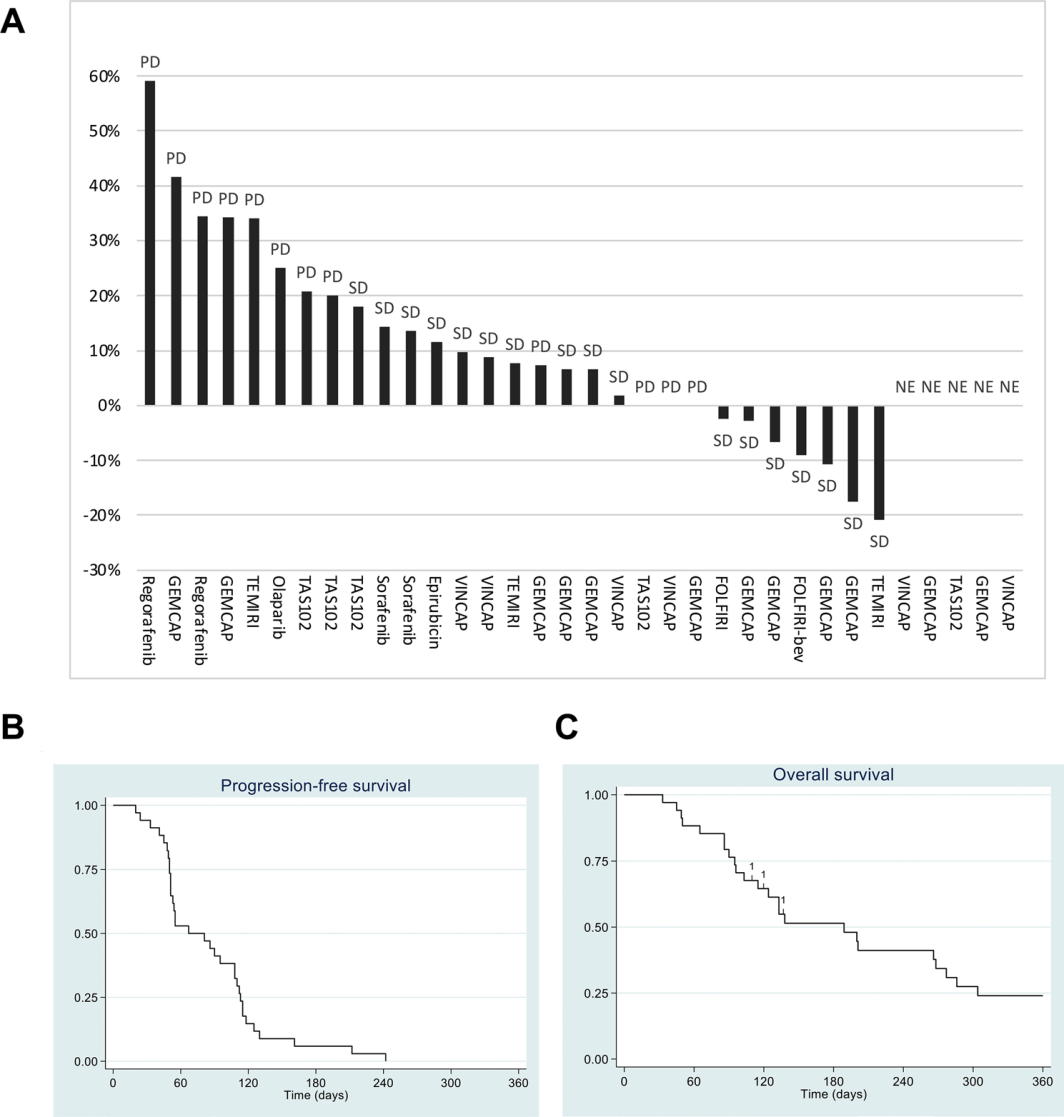
(**A**) The best response to systemic treatment during the treatment period in 29 patients with measurable disease and five patients with non-evaluable disease according to the Response Evaluation Criteria In Solid Tumors version 1.1 (RECIST 1.1): percentage change in tumor burden. PD=progressive disease, SD=stable disease, GEM=gemcitabine, CAP=capecitabine, TEM=temozolamide, IRI=irinotecan, VIN=vinorelbine, FOLF=5-flourouracil and leucovorin, bev=bevacizumab. NE=non evaluable disease: one had no measurable disease at baseline, and four had clinical progression or death before the first scanning evaluation). Kaplan-Meier curves showing progression-free survival (**B**) and overall survival (**C**) of all 34 patients in the precision cohort. No patients were censored for PFS, and five patients still alive at the date of analysis were censored (two after 360 days)

Seventeen patients (50%, 95% CI 32–68) met the primary endpoint (PFS at two months). Table 2 shows the number of patients with PFS at two months according to the treatment administered. Post-hoc analyses showed that more patients met the primary endpoint in *NRAS* (N = 3) or *BRAF* (N = 2) mutated cases than in *KRAS* mutated (N = 21) or all wildtype cases (p = 0.02). No radiological response was observed. The individual response to the given treatment for each patient was based on the change in sum of tumor diameter according to RECIST 1.1. Figure 2 A-B illustrates the best response for the individual patients and treatments. The median PFS and OS were 67 days (95% CI 51–108) and 189 days (95% CI 103–277), respectively (Fig. 2C-D). Five patients alive were censored for OS, and none of them were censored for PFS. Four patients who stopped treatment for reasons other than death or progression were followed with regular imaging tests until death. Febrile neutropenia and peripheral motor neuropathy were the most common adverse events (Supplementary Table S3) observed in our patients after treatment. Serious adverse events were reported 14 times among eight patients, all caused by events requiring hospitalization or prolonged hospitalization (Supplementary Table S4).

## Discussion

Several early-phase clinical trials have shown specific anti-cancer effects from more drugs than recommended by clinical guidelines (such as ESMO and NCCN), but the average effect is too low to be generally approved. Also, third-line drugs recommended in international guidelines have no substantial clinical benefit, and it is not known which patients will benefit from re-challenge with a previously given treatment. A standard for identifying the patients who may benefit from these drugs is lacking. Functional precision medicine based on patient-derived tumor organoids for drug sensitivity screening is a new platform with a robust preclinical rationale, but prospective and interventional evidence is scarce. To our knowledge, this is the largest prospective clinical study using TDOs and drug sensitivity screening for the treatment of metastatic colorectal cancer after standard treatments.

We demonstrated that precision oncology using a functional approach with organoids derived from the individual patient’s metastasis and in vitro sensitivity testing was feasible. The primary endpoint was met, as half of the patients (17 of 34) were without progression at two months compared to the predefined limit of at least 14 of 45. A limitation of the study was the non-randomized comparison with the control arm derived from two historic last-line phase III trials. Another limitation was the rate of success in generating TDOs. To improve the rate, others have suggested organizing biopsy sessions and introducing new methods [18], and we have shown that the number of TDOs correlates with the number of tumor cells and probably with the stiffness of the metastasis, whereas necrosis in and size of metastasis were not related [19]. The prospective evaluation of functional medicine in a clinical setting, the inclusion of standard drugs, older chemotherapy with a known but small effect in colorectal cancer, and newer drugs are the main strengths of our trial. No intervening treatment was allowed between biopsy and experimental treatment.

In several observational trials, patient-derived tumor organoids underwent drug screening with a subsequent correlation between observed sensitivity and clinical effect. Wang et al. [20] tested the predictive accuracy of TDOs for response in stage IV colorectal cancer treated with 5FU and irinotecan or oxaliplatin. In the pilot study of 30 patients (43 samples), the culture success rate was 70%, and in the blinded testing of 71 patients (96 samples) it was 80%. The authors concluded that TDOs are predictive of therapy response. The high success rate of 3D cell culture was likely due to treatment naïve tumors and large-size resected samples. In contrast, our success rate in generating TDOs was only 53.6% probably due to smaller biopsies from metastatic tissue after several lines of therapy.

Ooft et al. [21] conducted an observational study in 29 evaluable cases, to compare the response of TDOs at first-line and second-line treatment with 5FU, irinotecan, and oxaliplatin treatment. TDOs were predictive of response to irinotecan-based treatment but not to oxaliplatin. In treatment naïve rectal cancer patients undergoing chemoradiation, Yao et al. ^23^ showed a strong correlation between clinical and in vitro response to radiotherapy, 5FU, and irinotecan. Vlachogiannis et al. [22] established a biobank of TDOs, including those of 16 patients with heavily treated colorectal cancer. Tumor-derived organoids were screened in a panel of 55 drugs, and the in vitro sensitivity mimicked the effect observed in the clinic. Narasimhan et al. [23] cultured tumor cells from peritoneal metastasis of 28 colorectal cancer patients with success in 19 (68%), resulting in treatment change outside the trial in two patients, of which one presented partial response. We are only aware of one previous clinical trial prospectively testing TDOs to treat colorectal cancer patients. Ooft et al. [24] performed a single-arm study testing the sensitivity of five small molecule protein kinase inhibitors with different targets, albeit not regorafenib. Six patients were treated, but none of them were responsive to the treatment. The authors used response as the primary endpoint, but the response is usually not expected in the third line setting [15, 16]. Therefore, PFS might be considered a more relevant endpoint in this setting.

The clinical applicability of our functional approach could be argued against based on the challenges in the culture of biopsies from heavily pre-treated metastases and patients at risk of clinical deterioration. Ninety patients entered our trial, successful biopsies were obtained in 82, and only 34 received individualized treatment (37.8%). However, the much more widely used genomic approach results in a lower rate of treated patients. In a large trial with 6000 patients, molecular profiling was successful in 93% while 18% were offered treatment [25]. The functional approach may benefit from further refinement, but it is promising in relation to treatment suggestions. Future trials testing the effect of precision medicine should include both functional and genomic testing [26, 27]. The time to generate viable TDOs in sufficient amounts to perform the drug screening and the standardization of both procedures are still limitations to overcome [28, 29]. In our study, we used a cell culture medium with similar composition as previously reported to generate TDOs of colorectal cancer [30, 31] and liver metastases of colorectal cancer [32]. Considering that the number of TDOs was variable, we opted to perform the drug assays (at least six replicates) instead of the phenotypic and molecular characterization (which required many TDOs), which is a limitation of our study. Also, tumor-derived organoids as an individual model for cancer drug screening has a strong scientific rationale and preclinical background but is also limited by the lack of tumor microenvironment and immune cells [33]. Therefore, testing drugs targeting these components and their interplay with cancer cells will require assays specifically developed for this purpose.

In the daily clinic, several treatment decisions to be made with colorectal cancer patients might benefit from establishing TDOs and ex-vivo sensitivity data. Even though the effect of different standard chemotherapies is comparable, selecting the most effective first-line treatment may impact the overall outcome and increase the resection rate in potentially resectable cases. In the adjuvant setting, treatment is based on risk factors such as lymph node positivity. Tumor-derived organoids is an encouraging model for refining the selection of the best treatment for a specific patient.

In conclusion, this is the largest prospective, interventional clinical trial of last-line systemic therapy in colorectal cancer based on tumor-derived organoids. We showed improved clinical outcomes compared to that expected from the best supportive care alone. Although our study has limitations, including a one-arm design and a historical control, the findings herein reported provide valuable insights into the potential clinical utility of TDOs in guiding last-line systemic therapy in colorectal cancer.

## Electronic supplementary material

Below is the link to the electronic supplementary material.

Supplementary Material 1

Supplementary Material 2

## Data Availability

Data is reported in details. Further data is available upon academical request congregated to n>/=5.

## List of Abbreviations

PFS: Progression-free survival
CI: Confidence interval
TAS-102: Trifluridine tipiracil
TDOs: Patient-derived tumor organoids
3D: Three-dimensional
PS: Eastern Cooperative Oncology Group performance status
RECIST: Response Evaluation Criteria in Solid Tumors
CT: Computerized tomography scan
Hgb: Hemoglobin
FOLFIRI: Irinotecan and 5FU
FOLFOX: Oxaliplatin and 5FU
FOLFOXIRI: Irinotecan, oxaliplatin and 5FU
OS: Overall survival
IQR: Interquartile range
CTCAE: Common Terminology Criteria for Adverse Events
SAE: Serious adverse events
5FU: 5-fluorouracil or derivatives
