# Supplementary material for: Precision medicine applied to metastatic colorectal cancer using tumor-derived organoids and in-vitro sensitivity testing: a phase 2, single-center, open-label, and non-comparative study
Source: J Exp Clin Cancer Res. 2023 May 5;42:115. doi: 10.1186/s13046-023-02683-4 (PMC10161587; doi:10.1186/s13046-023-02683-4)
Supplement: Supplementary file 1 — Supplementary Material 1 [file 13046_2023_2683_MOESM1_ESM.docx]

| **Supplementary Table 1**. IndiTreat drug panels tested in patient-derived organoids from metastatic colorectal cancer. | | | | | |
| --- | --- | --- | --- | --- | --- |
| **Drugs** | **Panel 1** | **Panel 2** | **Panel 3** | **Panel 4** | **Panel 5*** |
| 5FU |  |  |  | **+** | **+** |
| Encorafenib + Binimetinib + Cetuximab |  |  |  | **+** |  |
| Encorafenib + Cetuximab |  |  |  | **+** |  |
| Epirubicin | **+** | **+** | **+** | **+** | **+** |
| FOLFIRI |  | **+** | **+** | **+** | **+** |
| FOLFIRI + Cetuximab |  | **+** | **+** |  | **+** |
| FOLFOX |  | **+** | **+** |  | **+** |
| FOLFOXIRI | **+** | **+** | **+** | **+** | **+** |
| Gemcitabine | **+** |  |  |  | **+** |
| Gemcitabine + 5FU |  | **+** | **+** |  |  |
| Irinotecan |  |  |  | **+** | **+** |
| Olaparib | **+** |  |  | **+** |  |
| Regorafenib | **+** | **+** | **+** |  |  |
| Sorafenib | **+** |  |  | **+** |  |
| TAS-102 | **+** | **+** | **+** |  | **+** |
| Temozolomide + Irinotecan |  |  | **+** |  |  |
| Vemurafenib+ cetuximab+ irinotecan |  |  |  | **+** |  |
| Vinorelbine | **+** |  |  |  |  |
| Vinorelbine + 5FU |  | **+** | **+** |  | **+** |

* Panel 5 was used to fill in the gaps. Drugs for screening were prioritized in cases with limited number of tumor derived organoids.

**Supplementary Table S2.** Drugs sensitivity tests performed on patient derived organoids from metastatic colorectal cancer and the selected treatment for each patient.

Please see attached EXCEL file.

| **Supplementary Table S3.** Reported adverse events attributable to the given treatment in the precision cohort of 34 patients. Grading was according to Common Terminology Criteria for Adverse Events (CTCAE) version 4. | | | | |
| --- | --- | --- | --- | --- |
| **Adverse Events** | **Grade** | | | |
|  | **0** | **1-2** | **3-4** | **N/A** |
| Performance Status* | 10 | 18 | 2 | 3 |
| Nausea | 14 | 14 | 0 | 6 |
| Vomiting | 19 | 9 | 0 | 6 |
| Stomatitis | 18 | 10 | 0 | 6 |
| Diarrhea | 22 | 5 | 1 | 6 |
| Constipation | 21 | 7 | 0 | 6 |
| Peripheral sensory neuropathy | 13 | 15 | 0 | 6 |
| Peripheral motor neuropathy | 27 | 1 | 0 | 6 |
| Palmar-plantar erythrodysesthesia syndrome | 18 | 10 | 0 | 6 |
| Pain | 11 | 16 | 1 | 6 |
| Fatigue | 9 | 18 | 1 | 6 |
| Febrile neutropenia | 28 | 0 | 0 | 6 |
| Infection | 19 | 5 | 5 | 5 |

* the worst reported day one in a cycle

| **Supplementary Table S4.** Serious adverse events (SAE) reported during the entire trial. Fourteen SAEs were reported in eight patients. | | |
| --- | --- | --- |
| **Serious Adverse Events** | **Grade** | **Cause** |
| Rash | 3 | Study drug |
| Infection | 3 | Cancer |
| Fever | 3 | Cancer |
| Novo virus | 3 | Other disease |
| Infection | 3 | Cancer |
| Progression | 3 | Cancer |
| Hydronephrosis | 3 | Cancer |
| Abdominal pain | 3 | Cancer |
| Constipation | 2 | Cancer |
| Constipation | 3 | Other treatment |
| Infection | 3 | Study drug |
| Obstruction of stent | 3 | Cancer |
| Ileus and infection | 3 | Cancer |
| Infection | 3 | Cancer |

**
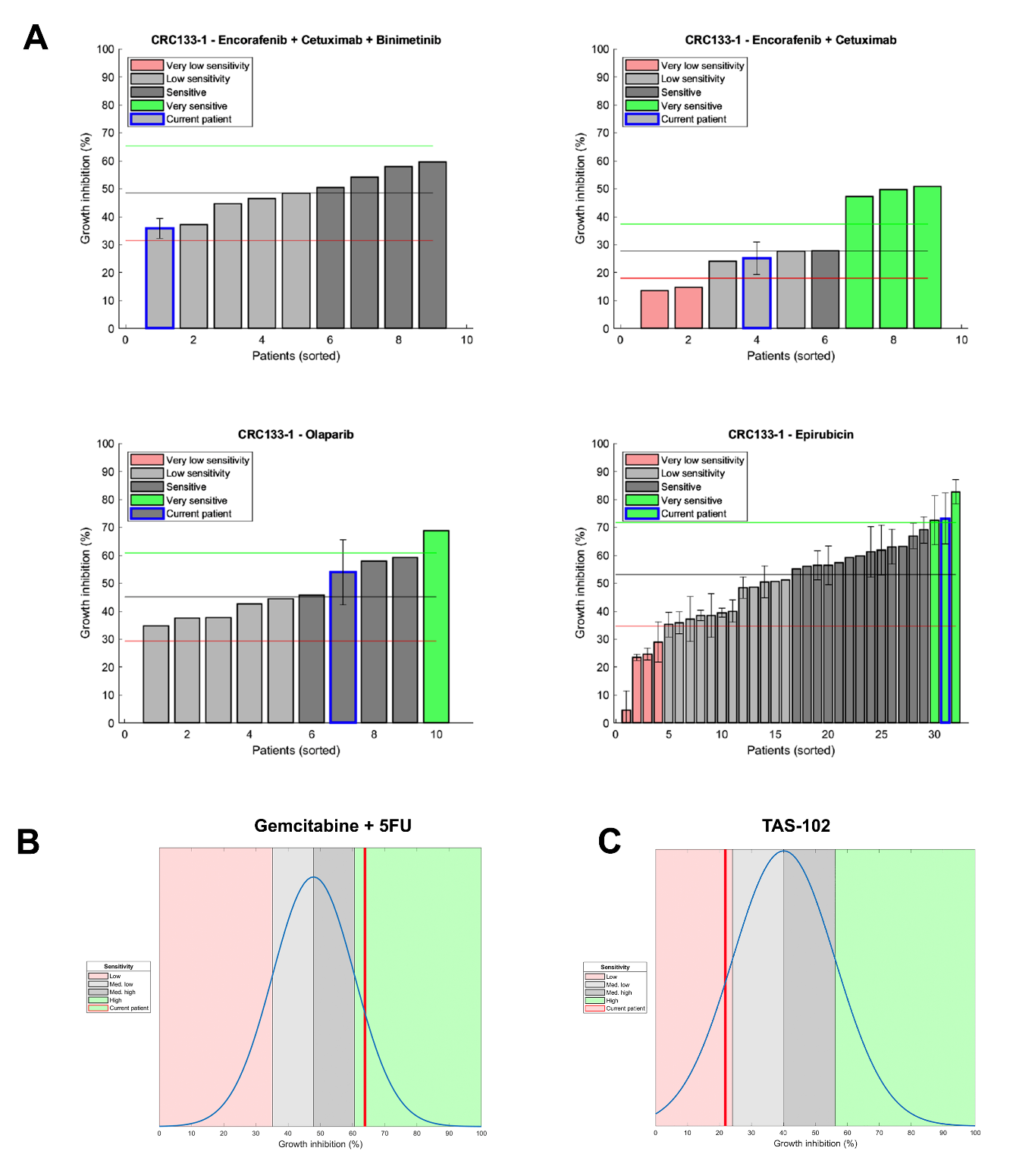
**

**Supplementary Figure S1**. (**A**) Representative examples of drugs response tested in tumor-derived organoids of one patient. The graphs marked by blue bars indicate the response of each drug in comparison with the response of sorted patients **(B)** Representative examples of a high response to Gemcitabine + 5-FU and a low response to TAS-102 observed in cells from metastatic colorectal cancer patients.

**
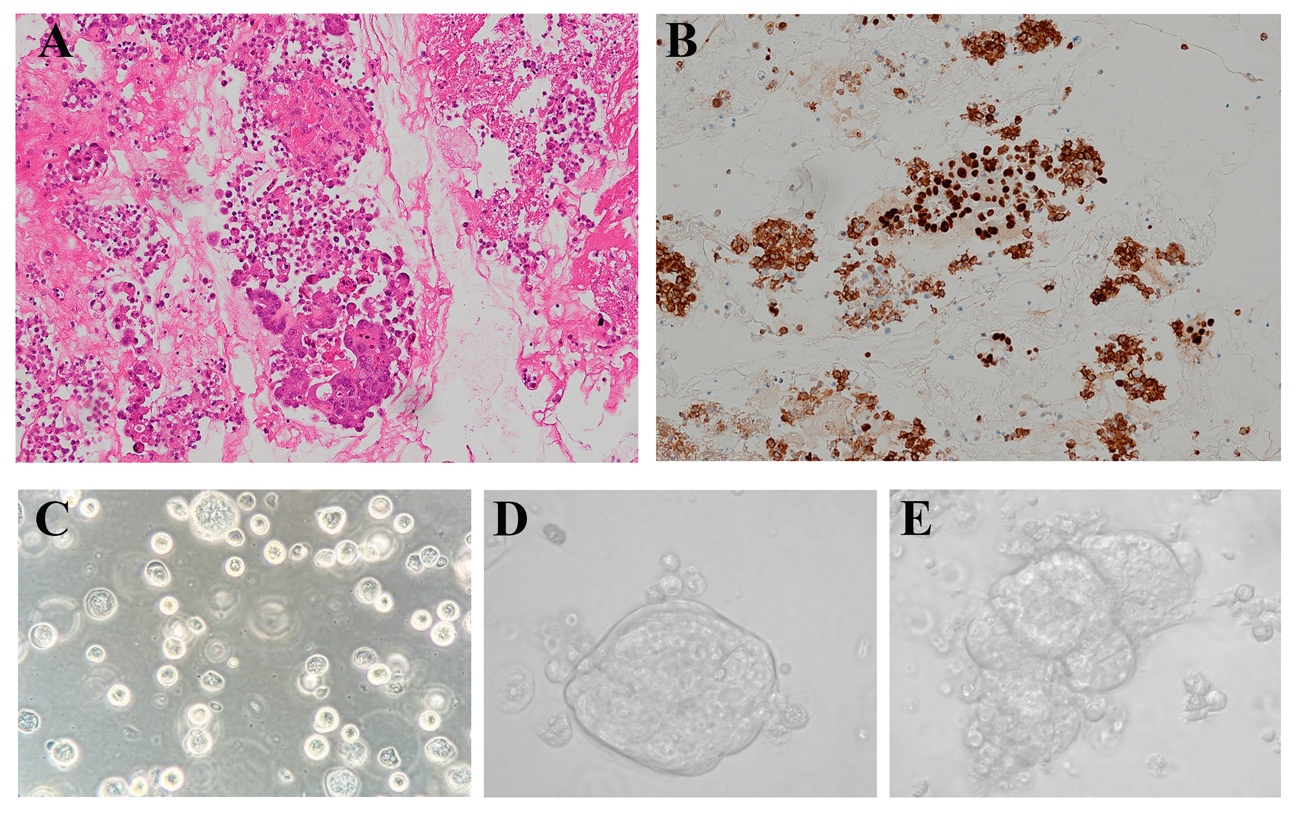
**

**Supplementary Figure S2. (A)** Hematoxylin-eosin section of a tumor-derived organoid at 100 times magnification with a cluster of epithelial cells arranged in an abnormal glandular formation. (**B**) Immunohistochemical staining for CDX2 (clone AMT 28, NovoCastra; 1:50) at 100 times magnification. The nuclear staining of the abnormal epithelial cells documents the colorectal origin. (**C-E**) Representative examples of tumor derived organoids from three pacients (C: 20X. D-E: 40X).
